# Sar1 localizes at the rims of COPII-coated membranes *in vivo*

**DOI:** 10.1242/jcs.189423

**Published:** 2016-09-01

**Authors:** Kazuo Kurokawa, Yasuyuki Suda, Akihiko Nakano

**Affiliations:** 1 Live Cell Super-Resolution Imaging Research Team, RIKEN Center for Advanced Photonics, 2-1 Hirosawa, Wako, Saitama 351-0198, Japan; 2 Laboratory of Molecular Cell Biology, Faculty of Medicine, University of Tsukuba, Tsukuba, Ibaraki 305-8575, Japan; 3 Department of Biological Sciences, Graduate School of Science, The University of Tokyo, Hongo, Bunkyo-ku, Tokyo 113-0033, Japan

**Keywords:** COPII-coated vesicle, ER, GTP hydrolysis, Sar1, Sec16

## Abstract

The Sar1 GTPase controls coat assembly on coat protein complex II (COPII)-coated vesicles, which mediate protein transport from the endoplasmic reticulum (ER) to the Golgi. The GTP-bound form of Sar1, activated by the ER-localized guanine nucleotide exchange factor (GEF) Sec12, associates with the ER membrane. GTP hydrolysis by Sar1, stimulated by the COPII-vesicle-localized GTPase-activating protein (GAP) Sec23, in turn causes Sar1 to dissociate from the membrane. Thus, Sar1 is cycled between active and inactive states, and on and off vesicle membranes, but its precise spatiotemporal regulation remains unknown. Here, we examined Sar1 localization on COPII-coated membranes in living *Saccharomyces cerevisiae* cells. Two-dimensional (2D) observation demonstrated that Sar1 showed modest accumulation around the ER exit sites (ERES) in a manner that was dependent on Sec16 function. Detailed three-dimensional (3D) observation further demonstrated that Sar1 localized at the rims of the COPII-coated membranes, but was excluded from the rest of the COPII membranes. Additionally, a GTP-locked form of Sar1 induced abnormally enlarged COPII-coated structures and covered the entirety of these structures. These results suggested that the reversible membrane association of Sar1 GTPase leads to its localization being restricted to the rims of COPII-coated membranes *in vivo.*

## Abstract (precis)

**Highlighted Article:** Detailed three-dimensional observations reveal that the localization of Sar1, which cycles on and off COPII-coated membranes, is restricted to the rims of the COPII-coated membranes *in vivo*.

## INTRODUCTION

Protein trafficking between organelles in eukaryotic cells occurs through membrane-bounded vesicles and carrier intermediates. Transport from the endoplasmic reticulum (ER) to the Golgi is mediated by coat protein complex II (COPII)-coated vesicles (Dancourt and Barlowe, 2010; Miller and Schekman, 2013). COPII-coated vesicles are concentrated at specialized sites within the ER, termed ER exit sites (ERES) or the transitional ER (Bannykh et al., 1996; Hammond and Glick, 2000; Orci et al., 1991). The key player for the formation of COPII-coated vesicles on the ER membrane is Sar1 GTPase (Nakano and Muramatsu, 1989) whose activation is initiated by GDP–GTP exchange catalyzed by the ER transmembrane guanine nucleotide exchange factor (GEF) Sec12 (Barlowe and Schekman, 1993). GTP binding by Sar1 induces its conformational change, which exposes an N-terminal amphipathic α-helix that then inserts into the ER membrane (Lee et al., 2005). Sar1-GTP on the ER membrane recruits Sec23 and Sec24 forming an inner layer on COPII-coated vesicles through the interaction with Sec23, and the transmembrane cargo is captured by the contact with Sec24 to form a pre-budding complex (Sato and Nakano, 2005). Sec13 and Sec31 proteins assemble into an outer layer on COPII-coated vesicles around the pre-budding complex through interactions with Sec23, and then inner and outer layers polymerize to form a caged structure (Lee et al., 2004; Stagg et al., 2006, 2008). The inner and outer layers have other functions; Sec23 is a GTPase-activating protein (GAP) for Sar1 (Yoshihisa et al., 1993), and this GAP activity is enhanced by Sec31 (Antonny et al., 2001). GTP hydrolysis by Sar1 in turn causes its dissociation from the membrane (Antonny et al., 2001).

Insertion of the N-terminal amphipathic α-helix of Sar1 into the membranes of synthetic liposomes deforms the liposomes, causing them to form tubules by generating membrane curvature (Bielli et al., 2005; Lee et al., 2005). These tubules are reported to form various structures, such as flexible tubules, beads-on-a-string-like constricted tubules and rigid tubules (Bacia et al., 2011; Long et al., 2010). Introducing a GTP-locked form of Sar1 alone into permeabilized mammalian cells can induce expansion of tubules from the ER membrane (Aridor et al., 2001; Bielli et al., 2005). Thus, activated Sar1 itself has an ability to deform the ER membrane. In addition, *in vitro* cell-free experiments with synthetic liposomes, proteoliposomes or in a planar lipid bilayer have shown that Sec23 and Sec24, Sec13 and Sec31, and Sar1-GTP together are sufficient for the formation of COPII vesicles, suggesting that the GTP-locked form of Sar1 is sufficient for COPII vesicle formation (Futai et al., 2004; Matsuoka et al., 1998; Sato and Nakano, 2004; Tabata et al., 2009). Nevertheless, multiple rounds of the Sar1 GDP–GTP cycle are required for efficient cargo concentration (Tabata et al., 2009). Furthermore, a GTP-locked mutant of Sar1 (yeast Sar1 H77L and mammalian Sar1 H79G; note mammalian Sar1 is also known as SAR1A) blocks cargo transport *in vivo* (Aridor et al., 1995; Saito et al., 1998). Therefore, the nucleotide-bound state of Sar1 must be spatially and temporally controlled to form COPII vesicles, to concentrate cargo into COPII vesicles, and to transport cargo to the Golgi complex *in vivo*. Here, we used super-resolution confocal live imaging microscopy (SCLIM) (Kurokawa et al., 2013) to document with high-resolution and high-speed the three-dimensional (3D) position and dynamics of Sar1 in living cells of the yeast *Saccharomyces cerevisiae*.

## RESULTS

### Sar1 distributes throughout the ER membrane and shows some accumulation around ERES

**Fig. 1.**
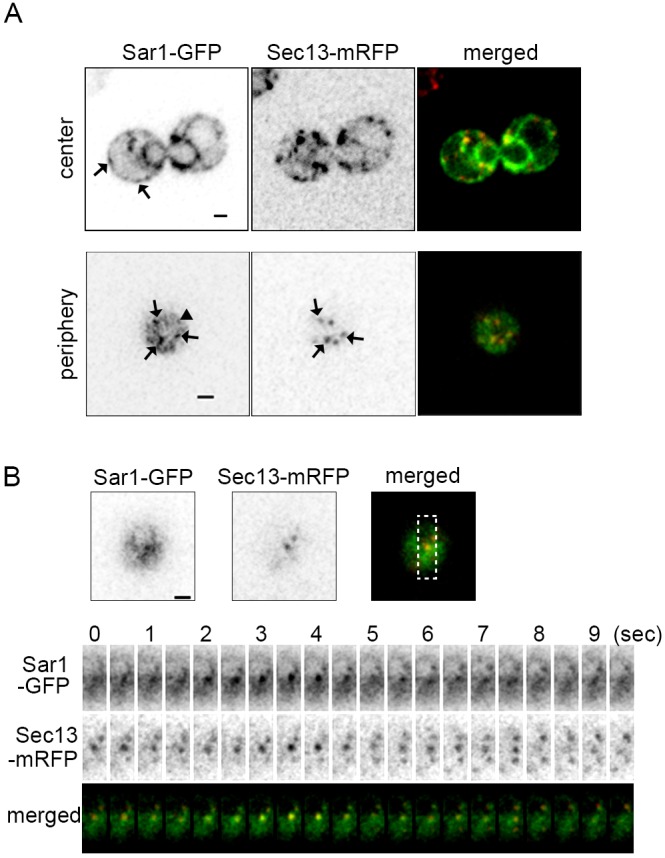
**Sar1 distributes throughout the ER membrane and shows some accumulation around ERES.** Wild-type cells expressing Sar1–GFP (green) and Sec13–mRFP (COPII outer coat, red) were observed with SCLIM. (A) We observed five independent cells (upper panels) and 12 independent cells (lower panels). Representative two-dimensional (2D) images near the center of a cell (upper panels) and at peripheral ER (lower panels) are shown. Sar1–GFP signal was distributed throughout the ER and appeared as punctate structures (arrows, upper left panel). These Sar1–GFP puncta were more clearly visualized in a single focal plane at the peripheral ER (arrows, lower left two panels). Most ERES colocalized with Sar1-GFP puncta (merged images). The arrowhead indicates Sar1–GFP puncta that did not colocalize with Sec13–mRFP. Scale bars: 1 μm. (B) We observed 15 independent cells. Representative 2D time-lapse images at the peripheral ER are shown. Sar1–GFP always showed a partial colocalization with Sec13–mRFP. The boxed region indicates the area shown in the lower panels. Scale bar: 1 μm.

In *S. cerevisiae*, ERES appear as numerous scattered puncta throughout the ER and are positioned at high-curvature domains of the ER membrane (Okamoto et al., 2012; Shindiapina and Barlowe, 2010). We used SCLIM to examine Sar1–GFP behavior around ERES in living yeast cells. Sar1–GFP had a complementary function to endogenous Sar1 (Fig. S1). Confocal imaging near the center of the cell indicated that Sar1–GFP was present on the nuclear envelope and the peripheral ER as previously observed in immunofluorescence studies using anti-Sar1 antibody (Nishikawa and Nakano, 1991; Rossanese et al., 1999). However, the distribution was not evenly continuous, and some punctate structures were seen (Fig. 1A, upper panels); these puncta reportedly colocalize with the peripheral ER membrane protein Sec16 (Yorimitsu and Sato, 2012). Similar Sar1–GFP puncta were more clearly visualized in a single focal plane at the peripheral ER (Fig. 1A, lower panels). Sec13–mRFP, a COPII outer coat protein, is used as an ERES marker, and simultaneous observation of Sar1–GFP and Sec13–mRFP indicated that most of the ERES colocalized with Sar1 puncta (Fig. 1A, arrows in lower panels, 96.7% of 64 ERES in 12 cells). Notably, some Sar1 puncta did not coincide with Sec13 (Fig. 1A, arrowhead in lower panels, 30.0% of 87 Sar1 puncta in 12 cells), and these Sar1 puncta might represent intermediates that are capturing cargo and/or forming COPII vesicles.

We next conducted simultaneous dual-color 2D time-lapse observation of Sar1–GFP and Sec13–mRFP. Sec13–mRFP showed a typical stable localization with some fluorescence signal fluctuation, as we have previously reported, suggesting that new COPII vesicles form at ERES and then bud and/or collapse at ERES (Fig. 1B) (Bevis et al., 2002; Kurokawa et al., 2014). Sar1–GFP puncta were mostly located near Sec13–mRFP; however, the overlap between Sar1–GFP and Sec13–mRFP signals did not occur over the entire ERES region; it was necessarily limited to the relatively small areas of Sec13–mRFP signal (Fig. 1B).

### Sar1 accumulates at the rims of COPII-coated membranes, but is excluded from the rest of the COPII-coated membrane

**Fig. 2.**
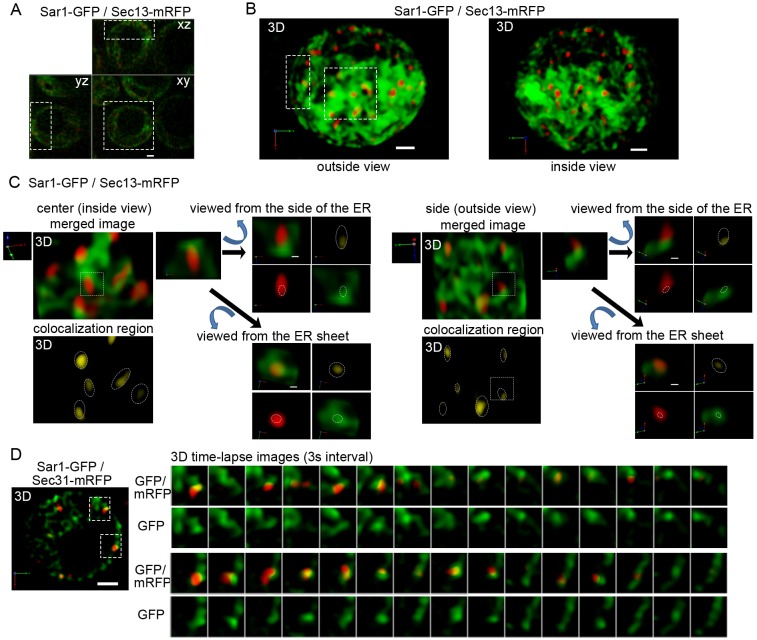
**Restricted localization of Sar1 at the rim of COPII-coated membrane.** Wild-type cells expressing Sar1–GFP (green) and Sec13–mRFP (COPII outer coat, red) were observed with SCLIM. Optical slices were taken 100 nm apart. (A) We observed 12 independent cells. Representative *xy*, *xz* and *yz* images of a single cell are shown. (B) 3D reconstructed images of a cell hemisphere (boxed area of A) viewed from both outside and inside of the cell. (C) Magnified images of Sar1–GFP and Sec13–mRFP from the boxed area in B are shown (upper panels). Regions of colocalization between GFP and mRFP fluorescent signals are also shown (lower panels). These regions are restricted to the rims of COPII-coated membranes. Dotted ellipses show the area of mRFP signals. Dotted squares in these magnified images are further enlarged. The merged image shows the colocalized region; the Sec13–mRFP image and the Sar1–GFP image are also shown individually. These images are viewed from the side of the ER membrane and from the ER sheets. Dotted ellipses on the colocalization region images show the area of mRFP signals. Dotted ellipses on the mRFP and GFP images show the colocalization regions. Sar1–GFP and Sec13–mRFP colocalization is restricted to the rim regions of COPII-coated membranes. (D) A total of 10 wild-type cells expressing Sar1–GFP (green) and Sec31–mRFP (COPII outer coat, red) were observed with SCLIM. Four optical slices were taken at 0.2 μm apart around the center of cell. Representative dual-color 3D reconstructed time-lapse images (boxed areas) are shown in the right panels. COPII-coated vesicles labeled with Sec31–mRFP grew repeatedly on the ER membrane where Sar1–GFP signal accumulated. Regions of colocalization between Sar1–GFP and Sec31–mRFP were restricted to the rims of COPII membrane. Scale bars: 1 µm (A,B,D), 200 nm (C).

Partial colocalization of Sar1 and Sec13 in 2D images suggested that Sar1 does not localize to the entire region of COPII-coated membranes. Thus, we next examined the detailed 3D localization of Sar1 and COPII markers *in vivo*. The apparent size of Sec13–mRFP patches varied from <100 nm to >300 nm, suggesting that either a single COPII vesicle or a cluster of COPII vesicles can form at the ERES (Fig. S2). 3D dual-color observation of Sar1–GFP and Sec13–mRFP demonstrated that Sar1 was present throughout the ER membrane network and that ERES spots marked with the COPII coat marker Sec13–mRFP were located on this membrane network (Fig. 2A,B; Movie 1), as we have previously shown using Sec13–GFP and mRFP–Sec12 (Okamoto et al., 2012). High-resolution 3D images of Sar1–GFP and Sec13–mRFP are shown in Fig. 2C. 3D images viewed from the side of the ER membrane revealed that Sar1–GFP and Sec13–mRFP showed restricted colocalization at the rim regions of COPII-coated membranes (Fig. 2C; Movies 2 and 3). With 3D dual-color time-lapse observation, Sec31–mRFP signals showed repeated increase and decrease at the ER membrane regions where Sar1–GFP signals accumulated. Colocalization of Sar1–GFP and Sec31–mRFP was observed at the boundary between the ER membrane and COPII-coated membranes (Fig. 2D). These results indicate that the membrane-bound GTP form of Sar1 is restricted to the rims of the COPII-coated membranes and is excluded from the other areas of COPII-coated membranes.

### Sar1 assembly around ERES depends on Sec16 function

**Fig. 3.**
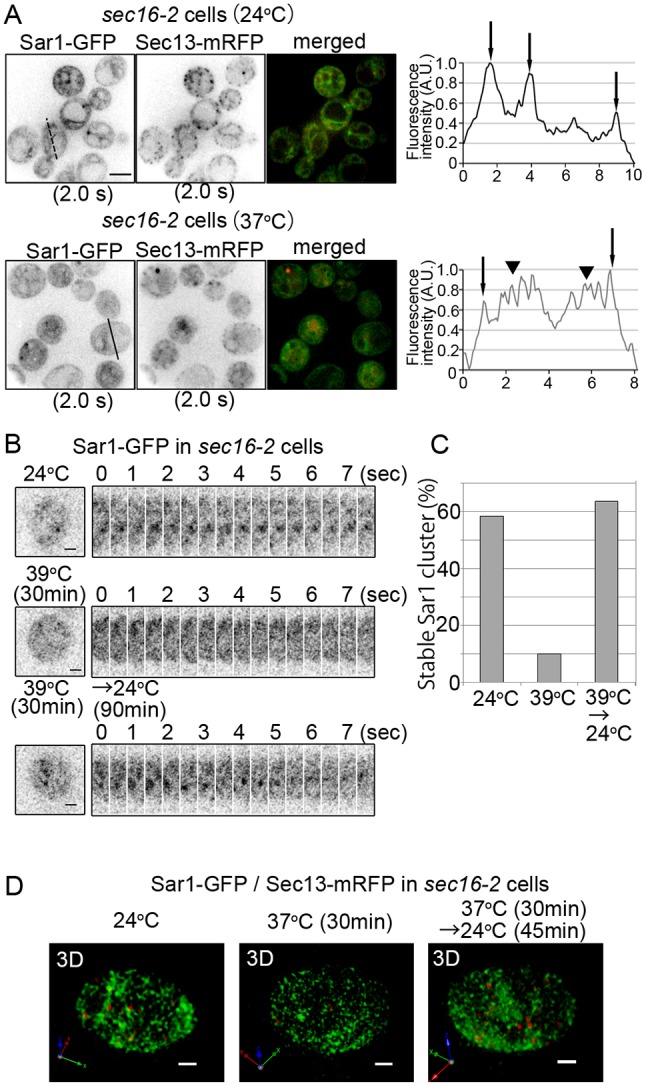
**Assembly of Sar1 around ERES depends on Sec16 function.** (A) *sec16-2* cells expressing Sar1–GFP and Sec13–mRFP were observed at the permissive (24°C) and the restrictive (37°C) temperatures. Representative 2D images (exposure time, 2 s) from the center of a cell are shown (upper panels). The intensity of GFP signals across a cell is also shown as a linescan (right panels). Arrows indicate the positions of the ER membrane (left and right arrows in upper panel, both arrows in lower panel) and the nuclear envelope (center arrow in upper panel); arrowheads indicate the position of the cytoplasm. Upon a shift to the restrictive temperature, the fluorescent signals from Sar1–GFP decreased in the ER membrane and increased in the cytoplasm. Sec13–mRFP dispersed in the cytoplasm. Scale bar: 5 μm. We conducted at least five independent experiments and representative images are shown. (B,C) At least 10 independent *sec16-2* cells expressing Sar1–GFP were observed. Representative 2D time-lapse images (exposure time, 0.5 s) at the peripheral ER are shown in B. Scale bar: 1 μm. Percentages of cells which have Sar1–GFP clusters stably localizing at their peripheral ER over 4 s are shown in C. Sar1–GFP clusters were evident in the cells cultured at the permissive temperature (24°C). Upon temperature-shift to the restrictive temperature (37°C) for 45 min, Sar1–GFP clusters on the peripheral ER disappeared. Cells were then shifted from 37°C back to 24°C and cultured for 90 min; after these 90 min, Sar1–GFP clusters were evident again. (D) We observed at least eight independent *sec16-2* cells expressing Sar1–GFP (green) and Sec13–mRFP (COPII outer coat, red). Representative 3D images were reconstructed from optical slices taken 0.1 µm apart. Upon temperature shift to the restrictive temperature (37°C), weak fluorescent signals from Sar1–GFP evidently localized to the ER membrane; however, COPII-coated vesicles labeled with Sec13–mRFP vanished from the ER membrane. Cells were then shifted from 37°C back to 24°C and cultured for 90 min; after these 90 min, COPII-coated vesicle formation and restricted colocalizaton between Sar1–GFP and Sec13–mRFP to the rim of COPII membrane were evident again. Scale bar: 2 μm.

We next examined the dynamics of Sar1 puncta around ERES. Previously, we have found that the expression of Sec12, the specific GEF for Sar1, does not overlap much with ERES in *S. cerevisiae* (Okamoto et al., 2012). Sec12 localization is distinct from the case of Sar1 observed in the present study. In collagen-secreting mammalian cells, concentration of Sec12 at ERES is only required for procollagen to exit the ER; it is not required for general protein secretion (Saito et al., 2014). Therefore, Sec12 is not a strong candidate for directing Sar1 accumulation around ERES. Another candidate that could modulate the localization of Sar1 is the ERES-localized protein Sec16, which interacts directly with Sar1 and has been implicated in ERES organization (Yorimitsu and Sato, 2012). *Drosophila* Sec16 specifically interacts with the GTP-bound form of Sar1, but not with the GDP-bound form (Ivan et al., 2008). Based on these reports, we examined Sar1 assembly around ERES in the cells mutant for the temperature-sensitive allele *sec16-2*. In *sec16-2*, a leucine residue is replaced by a proline residue at position 1089 (Shindiapina and Barlowe, 2010), and this mutant Sec16 protein is unable to localize at ERES at the restrictive temperature (37°C) (Yorimitsu and Sato, 2012). 2D dual-color observation near the center of *sec16-2* cells expressing Sar1–GFP and Sec13–mRFP at the permissive temperature (24°C) showed distinct peaks for membrane-bound Sar1–GFP signals on the nuclear envelope and the peripheral ER membrane (Fig. 3A, arrows in right panel). However, upon up-shift to the restrictive temperature, the Sar1–GFP signal was diffused throughout the cytoplasm (Fig. 3A, arrowheads in right panel) and Sar1–GFP clusters were markedly decreased (Fig. 3A). The number of Sec13 dots (ERES) was also substantially decreased at the restrictive temperature. These results indicate that Sar1 accumulation at ERES, and consequently COPII coat assembly, are severely impaired due to the loss of Sec16 function at ERES. The remaining few Sec13 dots still showed partial colocalization with Sar1–GFP, suggesting again that the Sar1 cluster has an important role in COPII-coated vesicle formation. 2D observation of Sar1–GFP at the peripheral ER of *sec16-2* cells also gave similar results. The Sar1–GFP cluster showed stable localization at the permissive temperature. Upon up-shift to the restrictive temperature, Sar1–GFP accumulation was defective (Fig. 3B,C). This defect was reversible and, after the down-shift to the permissive temperature, Sar1–GFP puncta reappeared and stably localized (Fig. 3B,C). We next conducted 3D dual-color observation of Sar1–GFP and Sec13–mRFP in *sec16-2* cells. As shown in Fig. 3D, at the restrictive temperature, the Sar1–GFP signals were reduced and were scattered throughout the cells; they appeared to be distributed randomly on ER membrane structures. COPII-coated regions labeled with Sec13–mRFP almost completely disappeared from the cells. Upon down-shift to the permissive temperature, COPII coat assembly (Sec13–mRFP spots) and restriction of Sar1 localization to the rims of the COPII-coated membranes were recovered (Fig. 3D). These results suggest that Sec16 at ERES is required as a scaffold for Sar1–GTP-dependent COPII coat assembly and is involved in the regulation of the level of Sar1–GTP at the rim regions of COPII-coated membranes.

### Restricted localization of Sar1 at the rims of COPII-coated membranes depends on GTP hydrolysis by Sar1

**Fig. 4.**
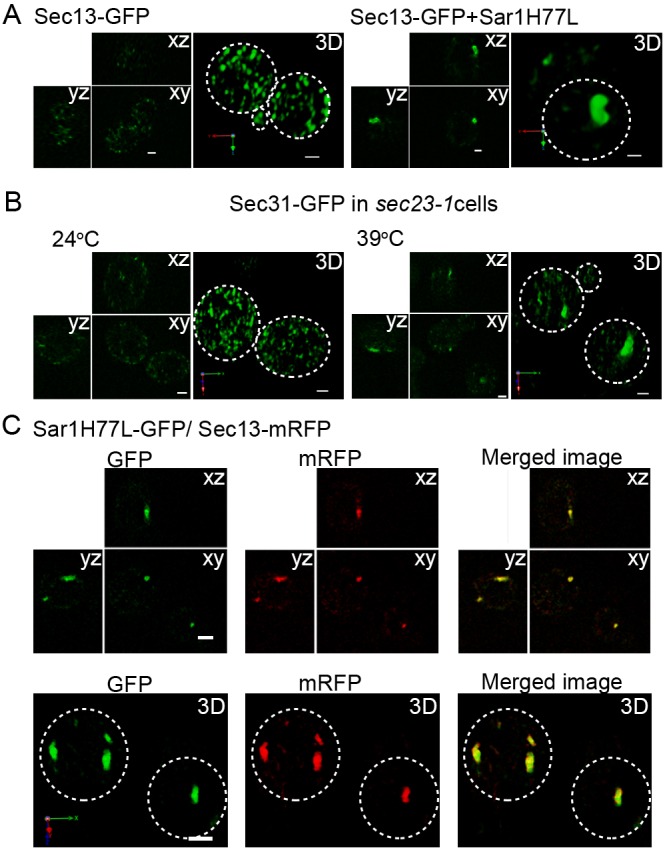
**Restriction of Sar1 localization** **to the rims of COPII-coated membranes depends on Sar1 hydrolysis of** **GTP.** (A) Wild-type cells expressing Sec13–GFP (COPII outer coat) with or without pGAL-Sar1H77L (GTPase-deficient mutant form) were cultured in galactose medium for 2 h at 24°C. Optical slices were taken 0.1 μm apart. We observed at least 12 independent cells and representative *xy*, *xz* and *yz* images, and 3D reconstructed images are shown. Expression of Sar1H77L induced formation of enlarged structures labeled with Sec13–GFP. (B) *sec23-1* cells expressing Sec31–GFP (COPII outer coat) were cultured at the permissive (24°C) and the restrictive (39°C) temperatures. Optical slices were taken 0.1 μm apart; *xy*, *xz* and *yz* images and 3D reconstructed images are shown. Abnormal elongated vesicular structures labeled with Sec31–GFP were observed in the cells that had been cultured for 60 min at the restrictive temperature. We observed four independent cells at 24°C and nine independent cells at 39°C. (C) A total of 14 independent wild-type cells expressing Sec13–mRFP (COPII coat, red) and Sar1H77L–GFP were observed. Sar1H77L–GFP is expressed under the control of the heat-shock promoter when cells were cultured at 37°C for 60 min. Optical slices were taken 0.1 µm apart, and representative *xy*, *xz* and *yz* images and 3D reconstructed images are shown. The GTP-locked form of Sar1–GFP accumulated on the entirety of these enlarged structures coated with COPII coat protein. Dotted circles in the images highlight the edges of cells. Scale bars: 1 μm.

On COPII-coated membrane, inner and outer coat proteins, Sec23 and Sec24, and Sec13 and Sec31, respectively, facilitate hydrolysis of GTP by Sar1; the hydrolysis then causes Sar1 to dissociate from the membrane (Antonny et al., 2001; Yoshihisa et al., 1993). We postulated that a mechanism underlying restricted localization of Sar1 to the rims of COPII-coated membranes involves limited Sar1–GTP hydrolysis that is promoted by COPII proteins on the surface of COPII-coated membrane. Expression of a GTPase-deficient mutant form of Sar1 (Sar1 H77L) caused abnormal aggregation of Sec13–GFP; this pattern of GFP signal was consistent with formation of enlarged COPII-coated structures (Fig. 4A). These structures were reminiscent of the vesicular-tubular clusters and the vesicular-necklace membrane structures containing COPII coat proteins that form in semi-intact mammalian cells expressing Sar1 H77G (Bannykh et al., 1996). In temperature-sensitive *sec23-1* mutant cells, in which the Sec23 GAP activity declines at the restrictive temperature, we observed a similar abnormal aggregation of Sec13–GFP upon up-shift to the restrictive temperature (Fig. 4B). We then performed dual-color 3D observation of the cells expressing Sec13–mRFP and GFP-tagged Sar1 H77L (Sar1H77L–GFP) (Fig. 4C). 3D images of Sec13–mRFP and Sar1H77L–GFP clearly showed that the GTPase-deficient mutant form of Sar1 localized over the whole region containing the enlarged Sec13–mRFP-positive structures without being restricted to the rims of these structures (Fig. 4C). Sar1H77L–GFP did not distribute throughout the bulk of the ER in these cells (Fig. 4C). These results indicate that GTP hydrolysis by Sar1 is required for restriction of Sar1 localization to the rims of COPII-coated membranes *in vivo*.

## DISCUSSION

In this study, our high-resolution 3D live observation with SCLIM clearly showed that Sar1 localized on the whole ER membrane and on the rim regions of COPII-coated membranes but was absent from COPII-coated membranes distant from the ER membrane *in vivo* (Fig. 2). Because the apparent size of Sec13–mRFP patches varied from <100 nm to >300 nm, suggesting that either a single COPII vesicle or a cluster of COPII vesicles can form at the ERES, these results suggest that Sar1 association is restricted to rims of individual single or multiple COPII-coated vesicles or that it surrounds membrane patches that include multiple COPII vesicles. Our high-resolution 3D images demonstrated that Sar1–GFP signals did not localize around the perimeter of the large Sec13–mRFP patch, therefore, these results suggested that Sar1 localized to the rim regions of individual single or multiple COPII-coated membranes. This restricted localization of Sar1 required GTP hydrolysis by Sar1 (Fig. 4); these findings indicate that the GTPase cycle of Sar1, which regulates not only cargo selection but also reversible membrane association of Sar1 itself, is spatially and temporally controlled on the membranes. Membrane-associated Sec12 recruits Sar1 to the ER membrane and exchanges GDP to GTP on Sar1, then Sar1-GTP recruits inner and outer coat proteins. Coat proteins assemble continuously to form a cage structure, which also contributes to and helps generate membrane curvature. The two-step process of coat assembly, in which the Sec23 GAP activity and the Sec31-mediated GAP stimulation enhance Sar1 hydrolysis of GTP, drives Sar1 dissociation from the membrane (Yorimitsu et al., 2014). By contrast, GAP activity toward Sar1-GTP is inhibited by the presence of Sec16, which localizes at ERES and modulates interactions between Sec31 and the Sec23–Sar1 complex (Kung et al., 2012; Yorimitsu and Sato, 2012). Thus, the correct balance of the supply of Sar1-GTP and the control of GTP hydrolysis by the Sar1 regulators, the activators Sec23 and Sec31, and the inhibitor Sec16, might lead to the restriction of Sar1-GTP localization to the rims of COPII-coated membranes. Consistent with these notions, the membrane-bound GTP form of Sar1 was decreased and Sar1 did not accumulate at ERES where the rims of COPII-coated membranes contacts with the ER membrane when the Sec16 function was compromised by growth of *sec16-2* mutant cells at the restrictive temperature (Fig. 3).When the GTP-locked mutant form of Sar1 was expressed, abnormally large COPII-coated membrane structures formed, and Sar1 covered the whole surface of these structures and did not remain on the bulk ER membrane (Fig. 4). Therefore, the continuous supply of Sar1-GTP from the ER membrane initiates COPII assembly at ERES where Sec16 is present, and the GAP activities of COPII coat proteins eliminate Sar1 by GTP hydrolysis from regions beyond the rims of COPII-coated membranes. Such spatial regulation of Sar1 GTP hydrolysis would lead to restricted localization of Sar1 to the rims of COPII-coated membranes. It remains unknown whether Sec16 localizes at the rims of COPII-coated membranes in *S. cerevisiae*. Sec16 in mammalian, *Drosophila*, and *Pichia* *pastoris* does not entirely colocalize with COPII coat proteins at ERES (Bharucha et al., 2013; Hughes et al., 2009; Ivan et al., 2008), suggesting that Sec16 might show the restrictive localization on the COPII-coated membranes. Recently, Glick and colleagues have reported that *P. pastoris* Sec16 restrains the Sar1 GTPase cycle and that loss of this restraining activity alters ERES structure by accelerating COPII coat turnover (Bharucha et al., 2013). Incorporating the localization and the function of Sec16, those authors have been proposed that *P. pastoris* Sec16 is restricted to the rims of COPII vesicles, where it stabilizes Sar1-GTP (Bharucha et al., 2013). Because *P. pastoris* has a small number of ERES (two to six per cell), and Sec12 and Sar1 accumulate at these ERES with COPII coats and Sec16 (Soderholm et al., 2004), spatial regulation of GTP exchange and GTP hydrolysis on Sar1 would be expected to be different from that in *S. cerevisiae*. Therefore, assessing the precise 3D spatial relationship between Sec16, Sar1 and COPII coats in *S. cerevisiae* will be a target of our future studies. Exclusion of the GTP-locked form of Sar1 from the bulk ER membrane suggested that the reversible membrane association of Sar1 contributed to the supply of Sar1-GTP to the next cycle of cargo selection and COPII-coated vesicle formation. Sar1 has recently been shown to form oligomers through its C-terminal domain, which can induce tubulation and constriction of membrane *in vitro* (Bacia et al., 2011; Hariri et al., 2014; Long et al., 2010). Thus, restricted localization of Sar1 at the rims of COPII membranes might contribute to vesicular geometry that drives membrane closure and also regulate vesicle scission *in vivo*.

The limited localization of Sar1-GTP at the rims of COPII-coated membranes provides another important condition for further transport. The GTP-locked form of Sar1 is sufficient for COPII vesicle formation *in vitro* (Futai et al., 2004; Matsuoka et al., 1998; Sato and Nakano, 2004; Tabata et al., 2009), but inhibits ER–Golgi cargo transport *in vivo* (Aridor et al., 1995; Saito et al., 1998). The sequential interaction of Sec23 with Sar1, the TRAPPI complex and Hrr25 reportedly plays a very important role in the directional transport from the ER to the Golgi (Cai et al., 2007; Lord et al., 2011). The TRAPPI complex binds to Sec23 on COPII-coated vesicles and then recruits and activates Ypt1. Active Ypt1 binds to the Golgi-membrane-localized long coiled-coil tethering protein Uso1; this binding precedes and drives the uncoating of COPII and the fusion between the vesicles and the Golgi membrane. Because the GTP-bound form of Sar1 competes with the TRAPPI complex for binding with Sec23, GTP hydrolysis by Sar1 on the COPII membrane must precede these sequential protein interactions to ensure the directionality of ER–Golgi traffic (Lord et al., 2011). We have recently reported that cis*-*Golgi cisternae in yeast cells frequently approach COPII-coated membranes of ERES and capture cargo there (Kurokawa et al., 2014). Through this ‘hug-and-kiss’ action, the cis-Golgi membrane is postulated to directly contact the COPII coat to induce the sequential protein interactions of Sec23 with Sar1 to the TRAPPI complex as described above. Sar1-GTP, which prevents the Sec23–TRAPPI interaction, must be removed from the COPII cage before coming into contact with the Golgi membrane. Positioning of Sar1-GTP at the rims of the COPII-coated membranes and its elimination from the rest of COPII membranes thus enables the triggering of the sequential reaction between the COPII and the Golgi membranes even before vesicles are released (Kurokawa et al., 2014). This mechanism has to be maintained by spatially and temporally controlled GTP hydrolysis. As mentioned above and detailed elsewhere (Kurokawa et al., 2013), our live imaging by SCLIM has space resolution beyond the diffraction limit, but it is not able to distinguish individual vesicles. Development of a new generation of SCLIM, which has even higher temporal and spatial resolution, is forthcoming, and we are hoping to visualize formation and capture of individual vesicles during hug-and-kiss interactions in future work.

## MATERIALS AND METHODS

### Yeast strains, plasmids and culture conditions

We used the yeast *S.cerevisiae* strain YPH499 (*MATa ura3-52 lys2-801 ade2-101 trp1-Δ63 his3-Δ200 leu2-Δ1*) for all the experiments in this paper. Yeast cells were grown in MCD medium [0.67% yeast nitrogen base without amino acids (Difco Laboratories Inc.), 0.5% casamino acids (Difco Laboratories Inc.) and 2% glucose] with appropriate supplements. For live imaging, cell cultures were grown to mid-log phase at 24°C. Strains expressing fluorescent-protein-tagged Sar1, Sec13, Sec31 or some combination thereof were constructed by a PCR-based method using pFA6a plasmids as template, which is described in the yeast GFP database at the University of California, San Francisco (Huh et al., 2003). The following primers were used: 5′-TTATGAGAAATGGTTATTTAGAGGCGTTCCAATGGTTATCTCAATATATTCCGATCCCCGGGTTAATTAA-3′ and 5′-CTGTTGAATTCATGTGAATGTCATATAAAGGGTATAGATGTATACGTCAAGAATTCGAGCTCGTTTAAAC-3′ for Sar1, 5′-TTTATGGAAGGAAAATCTTGAGGGTAAATGGGAACCCGCTGGTGAAGTTCATCAGCGGATCCCCGGGTTAATTAA-3′ and 5′-CTCATTTGCATTCTTTTTTCTTTTGAGATGTTTCATTTTAAATTCTTGATACTCTGAATTCGAGCTCGTTTAAAC-3′ for Sec13, and 5′-AACTGGCTGACAGGAGTGAAGAGGTTGATTGGCATAGCTGAAGCGACTTTGAATCGGATCCCCGGGTTAATTAA-3′ and 5′-AGAAAAAAACAAGGCCAATACGCCACTTTTTGTACTGAAAGTTTTGAGACTGAAGAATTCGAGCTCGTTTAAAC-3′ for Sec31). For 3D time-lapse observation, cells expressed Sec31–mRFP, which was constructed by a PCR-based method using pFA6a plasmids as template, and Sar1–GFP, which was expressed under the control of the *ADH1* promoter on the low-copy plasmid pRS316 (Sato et al., 2001). Sar1H77L, and Sar1H77L–GFP were expressed under the control of the *GAL4* promoter and a heat-shock promoter (2× *HSP*), respectively, from the low-copy plasmid pRS316 (Sato et al., 2001).

### Fluorescence microscopy

Cells were immobilized on glass slides using concanavalin A and observed with SCLIM (Kurokawa et al., 2013). SCLIM was achieved with a Olympus model IX-71 inverted fluorescence microscope with a UPlanSApo 100× NA 1.4 oil objective lens (Olympus, Japan), a high-speed spinning-disk confocal scanner (Yokogawa Electric, Japan), a custom-made spectroscopic unit, image intensifiers (Hamamatsu Photonics, Japan) with a custom-made cooling system, and two EM-CCD cameras (Hamamatsu Photonics, Japan) for green and red observation. To increase the spatial resolution, a magnification lens (4× or 10×) was put in the light path between the confocal scanner and the spectroscopic unit (final magnification, ×267 or ×667). For 3D images, we collected optical sections spaced 0.1 or 0.2 µm apart by oscillating the objective lens vertically with a custom-made piezo actuator (Yokogawa Electric) that oscillated in the *z*-axis position at a high-repetition rate (100 µm at 10–30 Hz) and a fine step (minimum movement is 0.05 µm apart). For 3D images of whole cells, we took ∼80 optical sections spaced 0.1 µm apart. For 3D time-lapse images, we took seven sequential optical sections spaced 0.2 µm apart around the center of cells. 3D images were reconstructed and deconvolved through point-spread functions optimized for a spinning-disk confocal scanner using Volocity software (Perkin Elmer, MA). MetaMorph software (Molecular Devices, CA) was used to view the time-lapse images, for analysis of fluorescence intensity and for movie construction. A thermo-controlled stage (Tokai Hit, Japan) was used to observe temperature-sensitive mutants.
